# Circulating tumour cells as an indicator of early and systemic recurrence after surgical resection in pancreatic ductal adenocarcinoma

**DOI:** 10.1038/s41598-020-80383-1

**Published:** 2021-01-18

**Authors:** Yejong Park, Hye Ryeong Jun, Hwi Wan Choi, Dae Wook Hwang, Jae Hoon Lee, Ki Byung Song, Woohyung Lee, Jaewoo Kwon, Su Hyeon Ha, Eunsung Jun, Song Cheol Kim

**Affiliations:** 1 Division of Hepatobiliary and Pancreatic Surgery, Department of Surgery, Asan Medical Center, AMIST, University of Ulsan College of Medicine, Seoul, Republic of Korea; 2 Biomedical Engineering Research Center, Asan Medical Center, Seoul, Republic of Korea; 3 Department of Convergence Medicine, Asan Institute for Life Sciences, University of Ulsan College of Medicine and Asan Medical Center, Seoul, Republic of Korea

**Keywords:** Gastrointestinal cancer, Tumour biomarkers, Medical research, Surgical oncology

## Abstract

Early recurrence in pancreatic ductal adenocarcinoma (PDAC) is a decisive factor in determining a patient's prognosis. We determined in our current study whether circulating tumour cells (CTCs) exist in the blood of PDAC patients and can be used as a predictor of recurrence patterns (i.e. time and site) after surgical resection. Between December 2017 and November 2018, the mononuclear cell layer was obtained from the peripheral blood of 36 patients diagnosed with PDAC. CTCs were then isolated using the CD-PRIME™ platform and detected via immunostaining. The patient records were analyzed to correlate these data with survival and recurrence patterns. Twelve patients were CTC-positive (33.3%) and showed a significantly frequent rate of systemic recurrence (distant metastases and peritoneal dissemination) (p = 0.025). On multi-variable logistic regression analysis, CTC positivity was an independent risk factor for early recurrence (p = 0.027) and for systemic recurrence (p = 0.033). In summary, the presence or absence of CTC in the blood of the patients with PDAC could help predict the recurrence pattern after surgery. PDAC patients with CTC positivity at tumour diagnosis should therefore undergo a comprehensive strategy for systemic therapy and active monitoring to detect possible early recurrence.

## Introduction

Pancreatic ductal adenocarcinoma (PDAC) is the most fatal cancer worldwide with a 5-year survival rate of less than 10%^1,2^. Only 20–30% of pancreatic cancer patients can undergo a curative resection despite recent technical progress in imaging modalities^2–4^. In addition, because of the early lymphatic and hematogenous spread from pancreatic cancers, local recurrences, distant metastases, and peritoneal seeding are frequent^5^. There is also a substantial rate of understaging, resulting in recurrence rates of approximately 20% within first 6 months after surgery^6^. The median disease-free survival is just over 1 year, rendering the benefit of surgery questionable for many patients^7–9^. Occult systemic disease is likely the main cause of early relapse, although there may be other contributing factors such as postoperative morbidity^10^. In addition, early recurrence is associated with a poor prognosis after curative intent surgery for PDAC^8,11,12^.

Effective prognostic biomarkers are important to optimize treatment, predict survival outcomes, and monitor strategies for patients with pancreatic cancer. Although there are several predictors and prognostic indicators for tumour recurrence and patient survival in pancreatic cancer, such as lymph nodes, tumour aneuploidy, carbohydrate antigen 19-9 (CA 19-9), tumour size, histological tumour differentiation, and tumour cell presence in resection margins, there is a need for additional biomolecular markers that can effectively predict disease progression^1,2,8,13^.

Circulating tumour cells (CTCs) identified in the peripheral blood are of interest as a diagnostic and prognostic indicator for systemic diseases including cancers^14,15^. CTCs originate from the tumour, are shed from the tissue into the bloodstream, and may be indicative of systemic dissemination^16^. Although the findings remain controversial^17,18^, overall survival is generally poor when CTCs are detected^4,5,13,15,19–21^. However, few studies to date have evaluated tumour recurrence rates or patterns in accordance with the detection of CTCs^18,22^.

Therefore, in the present analysis, we sought to determine whether the preoperative presence of CTCs is associated with the overall survival and recurrence-free survival in the patient with PDAC. In particular, we analyzed whether CTC detection in a PDAC cohort could be used as an indicator of early recurrence by analyzing the survival rates and recurrence patterns among these patients.

## Materials and methods

### Clinical characteristics of the study population

The present study was approved by the Institutional Review Board of Asan Medical Center (IRB No. 2017-1126). Between December 2017 and November 2018, 40 PDAC patients at our hospital provided written informed consent for CTC analysis of their peripheral blood. Patients with resectable or borderline resectable PDAC (n = 40) were enrolled consecutively based on preoperative imaging including computed tomography or magnetic resonance imaging. We collected and analyzed these data in accordance with the ethical standards of the Helsinki Declaration. After surgery in each case, a pathologist confirmed the diagnosis of PDAC from the surgical specimen in 36 patients. We excluded the remaining four patients from our analyses as they were confirmed with other tumours (distal common bile duct cancer in two patients, Ampulla of Vater cancer in one patient and intraductal papillary mucinous neoplasm in one patient).

Pancreaticoduodenectomy (PD), distal pancreatectomy (DP) or other procedures were performed in our study population in accordance with the tumour location and extension. The clinical, pathological, and survival data for these cases were collected from electronic medical records (EMRs) at our institution, retrospectively. The incidences of postoperative pancreatic fistula (POPF) and overall complications were assessed and graded on the basis of the International Study Group of Pancreatic Fistula criteria^23^ and Clavien-Dindo complication classification^24^, respectively. Tumour, node, and metastasis (TNM) staging was conducted in accordance with the eighth edition of the American Joint Committee on Cancer (AJCC) manual^25^. On the third or fifth day after surgical resection, all of the 36 study patients underwent CT to assess postoperative complications, including POPF. In addition, during their postoperative surveillance, CT and CA 19-9 levels were checked every 3 months during the first 2 postoperative years. If necessary, we conducted positron emission tomography (PET), magnetic resonance imaging (MRI) or biopsies to evaluate recurrence. When metastasis had been identified at the time of surgery, the pattern of tumour extension was checked whilst adjuvant chemotherapy was being performed.

We defined early recurrence as within 12 months of surgery, as described in previous studies^8,26^. Loco-regional recurrence indicated recurrence at the site of pancreatic resection^27^ and distant recurrence indicated recurrence at the liver, lungs, or any other distant site. Peritoneal carcinomatosis was defined as peritoneal dissemination. Systemic recurrence was defined in the present analysis as distant recurrence and peritoneal carcinomatosis.

### Isolation of the peripheral blood mononuclear cell layer from whole blood

Preoperative peripheral blood sampling was performed before incision in the operating room. For this, 7.5 mL of blood was collected from the enrolled patients before surgery in heparin tubes to prevent clotting and kept at 4 °C for processing within 1 h of collection. The inner surface of the conical tubes was coated with a coating solution (Clinomics, Ulsan, Republic of Korea) for 5 min. Ficoll-Paque PLUS (GE healthcare, Seoul, Republic of Korea) was then injected into the coated tube, and blood was slowly injected through the tube wall. The tubes containing Ficoll-blood samples were centrifuged at 800*g* (without acceleration or braking) for 15 min at room temperature (RT). The peripheral blood mononuclear cell (PBMC) layer was separated from the interface of the Ficoll-plasma layer through a density-gradient solution and then used for further CTC isolation^27^.

### Enrichment and enumeration of circulating tumour cells

The CD-PRIME™ platform (Clinomics, Ulsan, Republic of Korea), a centrifugation-force-based size-selective lab-on-a-disc for CTC isolation, was used for enrichment and enumeration of the patient CTCs^28,29^. The CD-PRIME™ kit consists of CD-CTC solo discs, all reagents and antibodies. The target CTCs in the blood sample were trapped on a membrane in a chamber on this semi-automatic platform on the basis of size selectivity. The PBMC layer isolated from whole blood was transferred to pre-coated conical tubes and mixed with enrichment solution. This mixed sample was then injected through the inlet of the CD-CTC solo disc in the CD-OPR-1000 operator and stored in the chamber of the disc. The disc was rotated in clockwise and anticlockwise directions to eliminate blood cells. At the end of enrichment step, the CTCs were trapped on the membrane in the chamber. At this step, after taking out the membrane, viable CTCs could be expanded for future studies. In this study, for enumeration of CTCs, the chamber was filled with the fixation solution for 20 min at RT.

The membrane with the fixed cells in the chamber was subsequently stained for CTCs and all buffers and antibodies were provided in the kit. Briefly, cells were incubated with the blocking solution for 20 min, and incubated with green and red fluorescence-labeled primary antibodies for EpCAM/pan-CK (CK 8, 18, and 19) and CD45 respectively for further 20 min. Cells were then washed by centrifugation of the disc in the operator. In every step, all reagents were added into the chamber of inlet in the disc. Finally, the cell-fixed and -stained membrane was taken out from the disassembled disc, placed on a glass slide, and mounted with the nucleic acid dye 4′, 6-diamidino-2-phenylindole (DAPI). CTC enrichment and staining were thereby completed within 120 min after blood collection.

To be considered a CTC, the cell should be round or oval and have a nucleus with positive staining for DAPI and EpCAM/CK and negative staining for CD45. The identification and verification of CTCs in our current study was performed independently by trained personnel under fluorescence microscope (Bioview, NesZiona, Israel) at a magnification of 100 ×. We defined the threshold for the detection of CTCs by using a cutoff of ≥ 1 CTC/7.5 mL of peripheral blood. In some cases, the cells showed positive staining for DAPI, CD45, and EpCAM/CK. As this was not consistent with the concept of "double-positive"^16,30–34^, we included them in the CTC-negative group.

### Statistical analysis

All data, depending on the type of variable, were presented as an absolute value, percentage, mean value with standard deviation (SD), or median with interquartile range (IQR). Statistical analysis was performed using the Student t-test for continuous outcomes with normal distribution, and the Mann–Whitney *U* test as a non-parametric test for continuous variables. For the binary outcomes, the χ^2^ test and Fisher exact test was used as the parametric and non-parametric test, respectively. Survival analysis and differences between survival estimates were performed using the Kaplan–Meier method with the log-rank test. In assessing the risk factors for early and systemic recurrence, only variables statistically significant in uni-variable analysis were included in multi-variable analysis, which was performed using logistic regression. All statistical analyses were performed using the Statistical Package for the Social Sciences (SPSS) version 21.0 (IBM Corp., Armonk, NY).

## Results

### Clinicopathological features and survival outcomes of the enrolled patients

We analyzed 36 patients with a PDAC confirmed by pathological examination after surgery (Table 1). The median age of these enrolled patients was 64 years (52–73) and 24/36 (66.6%) were men. The average CA 19-9 value was 48.6 U/mL (11.8–211.7), and 9/36 patients (25.0%) received neoadjuvant chemotherapy. Pathological examinations revealed that the body/tail was the most frequent tumour site (58.3%), the average tumour size was 2.7 cm (2.1–3.4), and that 18/36 cases (50.0%) had a lymph node metastasis. Postoperative complications, including POPF, were mostly absent or mild, and the average hospital stay was 9.5 days (8.0–12.8). 31/36 (86.1%) received adjuvant chemotherapy, and no deaths occurred within 90 days.

**Table 1:** Clinico-pathological characteristics according to the detection of circulating tumour cells. *ASA classification* American Society of Anesthesiologists physical status classification, *BMI* body mass index, *CA 19-9* Carbohydrate antigen 19-9, *CTCs* circulating tumour cells, *PD* pancreaticoduodenectomy, *DP* distal pancreatectomy, *AJCC* American Joint Committee on Cancer, *PNi* perineural invasion, *LVi* lymphovascular invasion, *CR-POPF* clinical-related postoperative pancreatic fistula, *IQR* interquartile range, *NA* not applicable.

Variable	Total number (%) or median	CTC-negative group (N = 24, 66.7%)	CTC-positive group (N = 12, 33.3%)	P-value*
Demographic features				
Age, years				
Median	64	66	61	0.969
IQR	52–73	53–72	52–74	
Sex				
Male	24 (66.7%)	17 (70.8%)	7 (58.3%)	0.479
Female	12 (33.3%)	7 (29.2%)	5 (41.7%)	
ASA				
1	3 (8.3%)	2 (8.4%)	1 (8.3%)	0.587
2	31 (86.1%)	20 (83.2%)	11 (91.7%)	
3	2 (5.6%)	2 (8.4%)	0 (0.0%)	
BMI (kg/m^2^)				
Median	22.4	22.7	21.9	0.798
IQR	21.4–24.1	21.3–24.3	21.3–25.2	
CA 19-9 (U/mL)				
Median	48.6	51.7	25.1	0.345
IQR	11.8–211.7	12.3–131.0	7.8–314.3	
< 37	17 (47.2%)	10 (41.7%)	7 (58.3%)	
≥ 37	19 (52.8%)	14 (58.3%)	5 (41.7%)	
Neoadjuvant therapy				
Yes	9 (25.0%)	7 (29.2%)	2 (16.7%)	0.685
**Pathologic findings**				
Tumour site				
Head	15 (41.7%)	12 (50.0%)	3 (25.0%)	0.151
Body/tail	21 (58.3%)	12 (50.0%)	9 (75.0%)	
Operation type				
PD	14 (38.9%)	11 (45.8%)	3 (25.0%)	0.371
DP	18 (50.0%)	10 (41.7%)	8 (66.7%)	
Others	4 (11.2%)	3 (12.5%)	1 (8.3%)	
Tumour size (cm)				
Median	2.7	2.7	2.8	0.521
IQR	2.1–3.4	2.0–3.3	2.2–4.1	
Differentiation				
Well	5 (13.9%)	4 (16.7%)	1 (8.3%)	0.197
Moderate	21 (58.3%)	15 (62.5%)	6 (50.0%)	
Poor	8 (22.2%)	3 (12.5%)	5 (41.7%)	
N/A	2 (5.6%)	2 (8.3%)	0 (0.0%)	
T stage^†^				
T1	8 (22.2%)	7 (29.2%)	1 (8.3%)	0.123
T2	22 (61.1%)	14 (58.3%)	8 (66.7%)	
T3	4 (11.1%)	1 (4.2%)	3 (25.0%)	
N/A	2 (5.6%)	2 (8.3%)	0 (0.0%)	
N stage^†^				
N0	16 (44.4%)	12 (50.0%)	4 (33.3%)	0.440
N1	15 (41.7%)	8 (33.4%)	7 (58.3%)	
N2	3 (8.3%)	2 (8.3%)	1 (8.3%)	
N/A	2 (5.6%)	2 (8.3%)	0 (0.0%)	
M stage^†^				
M0	33 (91.7%)	21 (87.5%)	12 (100.0%)	0.536
M1	3 (8.3%)	3 (12.5%)	0 (0.0%)	
AJCC stage^†^				
I	15 (41.7%)	11 (45.8%)	4 (33.3%)	0.392
II	15 (41.7%)	8 (33.4%)	7 (58.3%)	
III	3 (8.3%)	2 (8.3%)	1 (8.3%)	
IV	3 (8.3%)	3 (12.5%)	0 (0.0%)	
PNi				
Yes	28 (77.8%)	17 (70.8%)	11 (91.7%)	0.333
LVi				
Yes	15 (41.7%)	9 (37.5%)	6 (50.0%)	
R0 resection^‡^				
R0	28 (77.8%)	20 (83.4%)	8 (66.7%)	0.397
R1	5 (13.9%)	2 (8.3%)	4 (33.3%)	
R2	3 (8.3%)	2 (8.3%)	0 (0.0%)	
**Postoperative outcomes**				
CR-POPF^¥^				
Yes	1 (2.8%)	0 (0.0%)	1 (8.3%)	0.333
Complication^¥^				
No	22 (61.1%)	15 (62.5%)	7 (58.3%)	0.873
Gr 1–2	12 (33.3%)	8 (33.3%)	4 (33.3%)	
≥ Gr 3	2 (5.6%)	1 (4.2%)	1 (8.3%)	
Hospital stay (days)				
Median	9.5	9.5	10.5	0.482
IQR	8.0–12.8	8.0–12.3	8.2–15.0	
Adjuvant therapy				
Yes	31 (86.1%)	19 (79.2%)	12 (100.0%)	0.146
90-day mortality				
Yes	0 (0.0%)	0	0	> 0.999

*The p-values were calculated using the Student t-test or Mann–Whitney *U* test for continuous variables and the χ^2^ test or Fisher exact test for binary variables between the CTC-negative and CTC-positive groups.

^†^The T, N, and M stages were defined based on the American Joint Committee on Cancer (AJCC) 8th edition.

^‡^R0 and R1 were defined as a distance of < 1 mm and ≥ 1 mm from the tumour to the resection margin, respectively. R2 was defined as the presence of residual extrapancreatic metastatic disease and localized disease that was resected with a grossly positive margin.

^¥^Postoperative pancreatic fistula (POPF) and overall complications were assessed and graded on the basis of the criteria of the International Study Group of Pancreatic Fistula and the Clavien–Dindo complication classification, respectively.

Multi-variable analysis of overall survival in the 36 study patients identified recurrence within 12 months (hazard ratio [HR], 4.792; 95% CI 1.547–14.843; p = 0.007) and a TNM stage above 3 (hazard ratio [HR], 5.116; 95% CI 1.547–14.843; p = 0.013) as meaningful independent variables (Table S1). Moreover, upon evaluating the survival curve as an additional item for recurrence, it was confirmed that survival was significantly poorer in cases showing an early recurrence within 12 months (p < 0.001) and in the patients with systemic recurrence (p = 0.010) (Fig. S1).

### Isolation and detection of circulating tumour cells from whole blood

CTCs were isolated from the PBMCs of the study patients and identified by immunostaining (Fig. S2). The CTC results in our present patient series are summarized in Table 2. 12/36 (33.3%) were identified as CTC-positive (i.e. CD45−/EpCAM, CK+), and the number of confirmed CTCs varied from 1 to 4. Among them, the cases that only one CTC was found was the most frequent with seven cases. In accordance with our CTC findings, we divided our study cases into negative and positive groups. CTC-negative group is CD45 (+ or −)/EpCAM, CK− cells (24/36, 66.7%). We then compared the clinic-pathological characteristics of these groups but found no significant differences found (Table 1).

**Table 2:** Detected number of circulating tumour cells (CTCs) in the blood of each patient. (Among 36 patients, the CTC-positive group included patients with EpCAM/CK (+) and CD45 (−) cell (N = 12). The CTC-negative group included patients with EpCAM/CK (−) and CD45 (+ or −) cell (N = 24).

Patient no	Total no. of cells (DAPI (+), n/7.5 mL)	Expression	No. of CTCs (DAPI (+), n/7.5 mL)
		CD45/EpCAM, CK/DAPI	
1	4529	+/+/+	0
2	7678	+/−/+	0
3	3372	+/+/+	0
4	2574	+/−/+	0
5	1084	+/−/+	0
6	3360	+/−/+	0
7	2304	**−/+/+**	1
8	1851	**−/+/+**	4
9	2054	**−/+/+**	1
10	1197	+/−/+	0
11	2734	+/+/+	0
12	3593	+/+/+	0
13	13,434	**−/+/+**	1
14	2397	+/−/+	0
15	2448	**−/+/+**	1
16	2493	+/−/+	0
17	4036	+/−/+	0
18	3096	+/−/+	0
19	5688	**−/+/+**	1
20	3018	**−/+/+**	2
21	4879	**−/+/+**	3
22	5046	+/−/+	0
23	2029	+/−/+	0
24	3568	+/−/+	0
25	968	**−/+/+**	1
26	2695	**−/+/+**	2
27	1460	**−/+/+**	1
28	1854	+/−/+	0
29	2016	+/−/+	0
30	1634	+/−/+	0
31	1164	+/−/+	0
32	2180	**−/+/+**	4
33	2990	+/−/+	0
34	2211	+/−/+	0
35	1634	+/−/+	0
36	2438	+/+/+	0

### Recurrence patterns and overall survival outcomes according to the detection of circulating tumour cells

**Figure 1:**
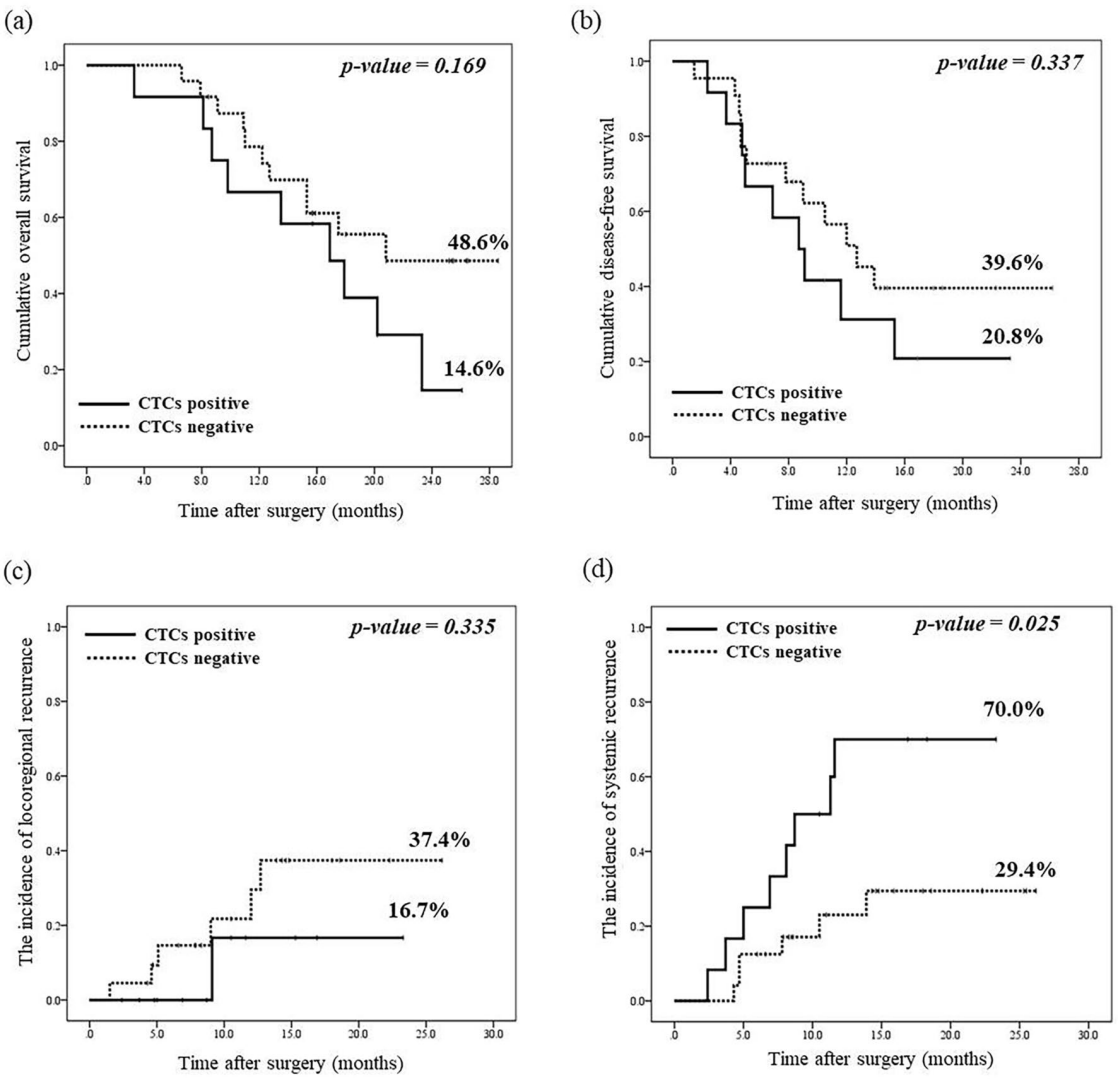
The survival and recurrence pattern according to the detection of circulating tumour cells (CTCs). CTC-positive group (n = 12), CTC-negative group (n = 24). (**a**) The cumulative overall survival according to the detection of CTCs after surgical resection. (**b**) The cumulative disease-free survival according to the detection of CTCs after surgical resection. (**c**) The incidence of loco-regional recurrence according to the detection of CTCs. (**d**) The incidence of systemic recurrence according to the detection of CTCs.

When we compared the presence or absence of recurrence in accordance with CTC detection, we observed that recurrence was slightly higher in the CTC-positive group (75.0%, 9/12) compared with the CTC-negative group (54.2%, 13/24) (Table 3). Further analysis was then conducted for the recurrence site and time of emergence (Table 3, Figure S3). There was no significant difference in 2-year overall survival between the CTC-positive and CTC-negative groups (14.6% vs. 48.6%, *p* = 0.169, Fig. 1a). The median survival time in the CTC-positive and CTC-negative groups were 17.6 months and 22.1 months, respectively. The 2-year disease-free survival and loco-regional recurrence did not differ between the two groups (*p* = 0.337,* p* = 0.335, Fig. 1b,c). The median recurrence times were 8.7 months and 12.7 months, respectively. In the CTC-positive patients, systemic recurrence had occurred more frequently than locoregional recurrence (p = 0.003, Fig. S3a). In the CTC-negative group, 8 out of 22 (36.4%) instances of recurrence were within 12 months, but all recurrences in CTC-positive patients occurred within 12 months (p = 0.031, Fig. S3b). However, when evaluating the cumulative recurrence according to the duration from surgery, it was confirmed again that systemic recurrence was significantly higher in the CTC-positive group (p = 0.025, Fig. 1d). Finally, we confirmed using multi-variable logistic regression analysis that CTC positivity is an independent risk factor for both early (odds ratio [OR], 8.770; 95% CI 1.275–60.346; p = 0.027) and systemic recurrence (odds ratio [OR], 5.600; 95% CI 1.146–27.370; p = 0.033) (Table 4).

**Table 3:** Subanalysis of the recurrence patterns according to the detection of circulating tumour cells.

Variable	Total number (%) or median	CTC-negative group (N = 24, 72.2%)	CTC-positive group (N = 12, 33.3%)	P-value*
Recurrence				
No	12 (33.3%)	9 (37.5%)	3 (25.0%)	0.378
Yes	22 (61.1%)	13 (54.2%)	9 (75.0%)	
N/A	2 (5.6%)	2 (8.3%)	0 (0.0%)	
Recurrence site				
Loco-regional	9 (40.9%)	7 (53.8%)	1 (11.1%)	0.003^†^
SMA	7	7	0	
Pancreas	1	0	1	
Mesentery	1	1	0	
Distant	7 (31.8%)	6 (46.2%)	2 (22.2%)	
Lung	1	1	0	
Liver	5	4	1	
Stomach	1	0	1	
Peritoneal carcinomatosis	6 (27.3%)	0 (0.0%)	6 (66.7%)	
Recurrence time				
Within 12 months				
Yes	17 (50.0%)	8 (36.4%)	9 (75.0%)	0.031
Follow-up duration (months)				
Median	15.6	15.2	17.6	0.651
IQR	8.8–19.3	10.9–22.3	8.3–19.3	

*CTCs* circulating tumour cells, *SMA* Superior mesenteric artery, *IQR* interquartile range.

*The p-values were calculated using the Student *t*-test or Mann–Whitney *U* test for continuous variables and the χ^2^ test or Fisher exact test for binary variables between the CTC-negative and CTC-positive groups.

^†^Systemic recurrence (distant metastasis and peritoneal carcinomatosis) vs. Loco-regional recurrence.

**Table 4:** Uni- and multi-variable logistic regression analysis for risk factors in early and systemic recurrence.

Variable	Uni-variable			Multi-variable		
	OR*	95% CI	P-value^†^	OR^1^	95% CI	P-value^†^
Risk factors for the early recurrence						
ASA						
< 3	1.000 (reference)		0.518			
≥ 3	2.286	0.187–27.994				
Neoadjuvant chemotherapy						
No	1.000 (reference)		0.698			
Yes	1.354	0.293–6.261				
Adjuvant therapy						
No	1.000 (reference)		0.176			
Yes	4.923	0.489–49.611				
CA 19–9(U/mL)						
< 37	1.000 (reference)		0.737			
≥ 37	1.266	0.329–4.867				
CTCs						
Negative	1.000 (reference)		0.038	1.000 (reference)		0.027
Positive	5.250	1.093–25.211		8.770	1.275–60.346	
R0 resection^‡^						
Yes	1.000 (reference)		0.051			
No	3.046	0.998 ~ 9.300				
Differentiation						
Well/moderate	1.000 (reference)		0.034			
Poor	11.200	1.193–105.132				
Perineural invasion						
No	1.000 (reference)		0.518			
Yes	2.308	0.187–27.994				
LVi						
No	1.000 (reference)		0.020	1.000 (reference)		0.027
Yes	5.958	1.332 ~ 26.662		8.165	1.277 ~ 52.225	
LN metastasis						
< 2	1.000 (reference)		0.034			
≥ 2	6.667	1.151–38.598				
Risk factors for the systemic recurrence						
ASA						
< 3	1.000 (reference)		0.992			
≥ 3	0.989	0.128 ~ 7.628				
Neoadjuvant chemotherapy						
No	1.000 (reference)		0.573			
Yes	1.398	0.437–4.478				
Adjuvant therapy						
No	1.000 (reference)		0.262			
Yes	28.117	0.083 ~ 66.339				
CA 19-9 (U/mL)						
< 37	1.000 (reference)		0.718			
≥ 37	0.824	0.289–2.354				
CTCs						
Negative	1.000 (reference)		0.020	1.000 (reference)		0.033
Positive	6.000	1.319–27.287		5.600	1.146–27.370	
R0 resection^‡^						
Yes	1.000 (reference)		0.044			
No	10.556	1.070–104.114				
Differentiation						
Well/moderate	1.000 (reference)		0.164			
Poor	1.470	0.854–2.530				
Perineural invasion						
No	1.000 (reference)		0.665			
Yes	0.753	0.209–2.714				
Lymphovascular invasion						
No	1.000 (reference)		0.159			
Yes	2.150	0.742–6.234				
LN metastasis						
< 2	1.000 (reference)		0.625			
≥ 2	1.707	0.200–14.576				

*ASA classification* American Society of Anesthesiologists physical status classification, *CA 19-9* Carbohydrate antigen 19–9, *CTCs* circulating tumour cells, *LN* lymph node, *CI* confidence interval.

*Odds ratios estimated using logistic regression analysis excluding possible confounding variables.

^†^The p values were calculated using the logistic regression analysis.

^‡^R0 was defined as a distance of the tumour from the resection margin < 1 mm.

**Figure 2:**
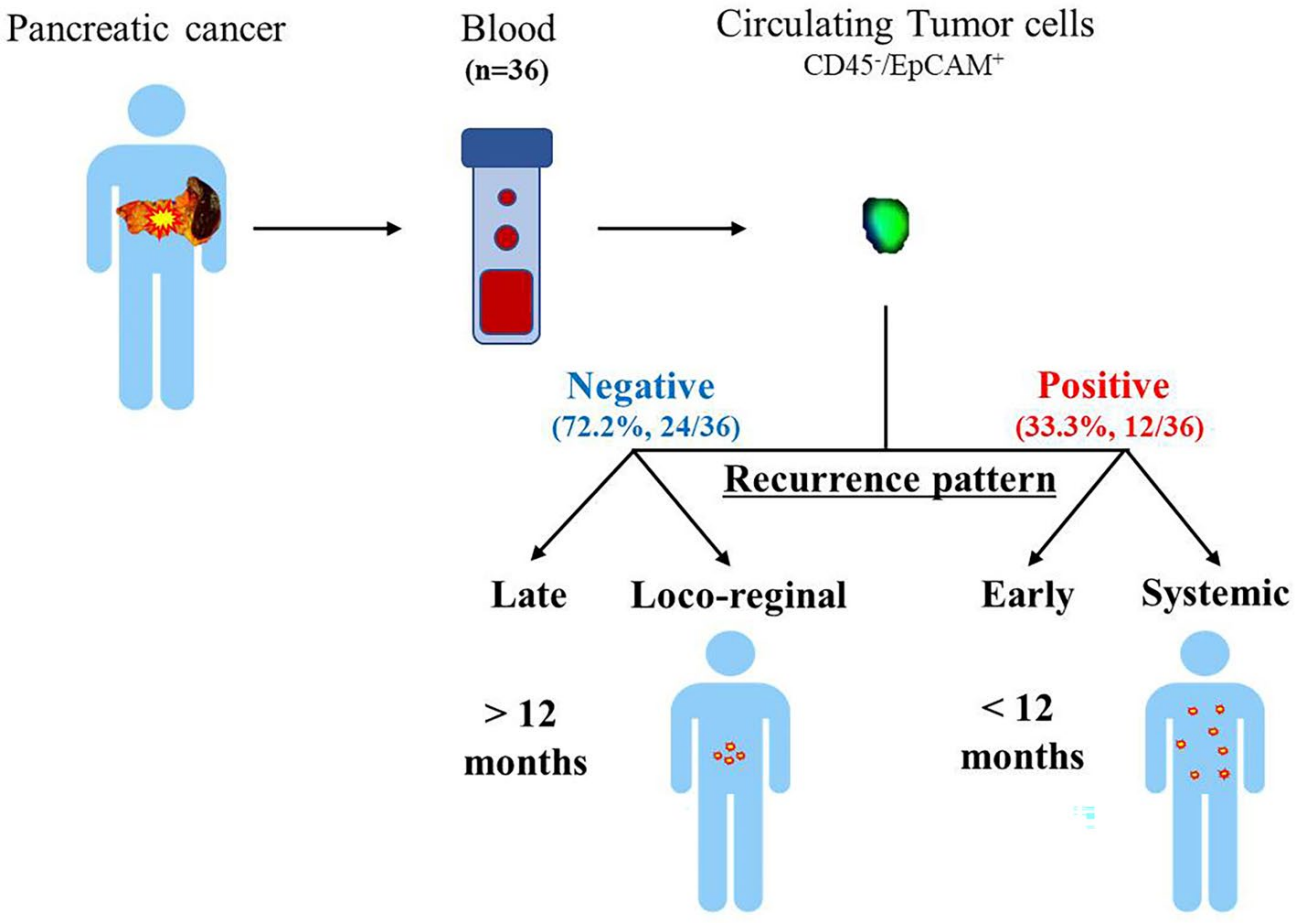
Postoperative recurrence pattern according to the presence of CTC in the blood of patients with pancreatic cancer. This graphic abstract focuses on the contents of Table 3. When CTC is detected before surgery, there is a tendency to have more early and systemic recurrence patterns.

Based on the results, we summarized the relationship between preoperative CTC detection and postoperative recurrence patterns (Fig. 2). If the CTC is not detected before surgery, the recurrence period is relatively late, and even if it recurs, there is a high possibility of loco-regional recurrence. On the other hand, when CTC is confirmed, it tends to show patterns of early and systemic recurrence.

## Discussion

We here demonstrate the clinical significance of CTCs for predicting the recurrence in pancreatic cancer patients. Liquid biopsies are now a research tool for clinical diagnosis and predicting prognosis. In the field of predicting tumour biology have proved valuable in clinical cancer research and therapeutics. In pancreatic cancer, which is very difficult to detect at an early stage, a liquid biopsy has considerable utility and it is a very useful tool for establishing the appropriate therapeutic protocol through the diagnosis and disease progression of the tumour. Liquid biopsy enables the detection of biomolecular markers to predict the disease progression such as microRNA, ctDNA, CTC, and exosomes^35^. Among these factors, CTCs can provide information regarding tumour features and disease status in clinical and research settings, indicating that their detection can be a useful biological tool^26,36–38^.

The CTC detection platforms used by researchers to date have varied. CTC detection was initially conventionally performed using immunomagnetic enrichment^20^, but technical advancements have further increased the specificity and accuracy of testing for these cells and analysis methods for CTCs continue to be developed. A well-known method is the commercially available CellSearch technique utilizing an anti-EpCAM antibody that is an FDA-approved immunoaffinity-based method for breast, prostate, and colorectal cancers^30,39^. In comparing our present method with CellSearch epithelial system, we found that the size-based CD-PRIME™ platform has advantages in its application of enrichment of viable cells within 30 min^40^. The CD-PRIME™ system is non-EpCAM-based enrichment of CTCs and enriched CTCs could be further analyzed in in-situ experiment for enumeration of CTCs^41^. The enumeration step is marker-dependent. In previous study, we used EpCAM, CK, and CD45 as conventional markers for enumeration of CTCs. The antigen-based CellSearch system however uses non-viable cells that cannot be used for subsequent analysis^42^. In addition, size-based CTC isolation (> 76%) has a higher detection rate than immunoaffinity based method (71%) but contamination with similar sized leukocytes remains a challenge to overcome with this approach^39,43,44^. Size-based method of CD-PRIME™ enables subsequent expansion of viable CTCs for culturing CTCs before immunofluorescence staining^45^. Though there are hurdles to elimination of blood cells except CTCs, enriched CTCs by CD-PRIME™ could be expanded by specified cell culture, it would be useful tool for further investigation of CTCs.

The detection rate of CTCs in our current study was 33.3%. Previous studies have reported rates of 11–93% and this difference was probably due to the detection methods and the disease status of the patients^5,17,26,46^. Iwanicki et al. reported that CTCs could be detected in 18 of 27 patients with advanced stage cancer (66.7%)^46^. Soeth et al. reported however that CTCs could be detected in only 7 of 27 patients with stage I-II disease out of a cohort of 154 cases^5^. In the study of Z’graggen et al. CTCs were detected in 53 of 72 patients with a primary PDAC (75%)^17^. Compared to previous studies, our study showed a somewhat lower detection rate (33.3%, 12/36) because of the low proportion of both patients with advanced stage tumours and cases receiving neoadjuvant chemotherapy in our population. It is noteworthy that 83.3% (30/36) of the patients included in our study had AJCC stage I/II disease, which are early stage tumours compared to those in the cohorts from prior reports. Furthermore, a previous study found that the CTC detection rate decreased after chemotherapy with 5-fluorouracil (80.5% before chemotherapy vs. 29.3% after chemotherapy)^47^. We found from our present analyses, after excluding patients who received neoadjuvant chemotherapy (n = 9), that the CTC detection rate increased slightly to 37.0% (10/27). Recently, a prospective study of pancreatic cancer patients reported that the number of CTCs in the blood was decreased by chemotherapy and surgery, which may be the basis that the number of CTCs can reflect the tumor burden^26^.

In the current study, CTC detection is limited for detection of EpCAM-low epithelial CTCs and mesenchymal CTCs. As these limitations, in case of epithelial CTCs, we would expect that pan-CK staining compensate the EpCAM low CTCs. Notwithstanding EpCAM/CK staining, total CTCs should also be stained for epithelial-mesenchymal transition markers because “Circulating” tumour cells are evading tumour cells originated from primary tumour tissue^26,48^. These cells might more likely be characterized for mesenchymal CTCs. Considering the association with CTCs and recurrence in this study, we thought that CD-PRIME™ platform with staining for traditional mesenchymal cancer markers (e.g. vimentin, twist) will be capable for increased precise detection of CTCs by in situ multiplex staining for distinguishing epithelial and mesenchymal cancers.

In our cohort, there was no significant difference in 2-year overall survival and disease -free survival between the CTC-positive and CTC-negative groups (*p* = 0.169,* p* = 0.337, Fig. 1). However, we secured meaningful results for the difference between the two groups through further analysis of the time and place of recurrence. In our CTC-positive group, early recurrence (i.e. within 12 months post-operatively) and systemic recurrence (distant and peritoneal carcinomatosis) were significantly frequent (Table 3, Fig. S3). Moreover, multi-variable logistic regression analysis (Table 4) indicated that CTC-positivity is a risk factor for both early (p = 0.027) and systemic recurrence (p = 0.033). Although there are abundant reports on the association between CTCs and survival^4,5,13,15,19–21^, few studies have evaluated the association between the recurrence rate or pattern and the detection of CTCs^18,22^. Mataki et al. reported that CTCs were detectable in only 6 of 20 patients, among whom five (80%) showed recurrence and one developed liver metastasis within 6 months post-operation. In contrast, only two patients (14.3%) in the CTC-negative group in their study showed recurrence^22^. Bissolati et al. reported that patients with CTCs in the portal vein had a higher incidence of liver metastases at 2 and 3 years after surgery (57.1% and 8.3%, respectively; p = 0.038)^18^. In that report, 36 patients were analyzed to confirm the tendency of recurrence within 12 months after surgery, and their median duration of follow-up was 15.6 months.

In this cohort, CTC positivity was significantly associated with both early and a systemic pattern of recurrence, but not associated with overall survival. These may be related to the limitation of our study with low numbers of cases and short duration for following up. To increase the clinical significance of CTCs for cancer patients and validate the diagnostic potential of CTCs, the use of multiple antibodies is one of important factor to increase sensitivity and minimize loss of CTCs followed by larger-scale studies with longer period. Ultimately, from the perspective of CTCs primarily shedding from tumour tissue, it is also important to analyse EMT/cancer stem cell markers and KRAS mutation status of CTCs besides considering tumour location. We hope that these results will give further information for CTC research of pancreatic cancer.

## Conclusions

CTCs are associated with early and systemic recurrence of PDAC. If CTCs are proven to be predictive of tumour extension or recurrence, we believe that their detection will be very helpful in determining initial treatment options in PDAC patients. Moreover, even after treatment, this detection will be helpful in determining the direction of systematic therapy through more active monitoring. We plan to conduct future patient-monitoring studies using advanced CTC detection techniques to further determine the most appropriate therapeutic strategies for PDAC.

## Supplementary Information

Supplementary Information.
