# Supplementary material for: Circulating tumour cells as an indicator of early and systemic recurrence after surgical resection in pancreatic ductal adenocarcinoma
Source: Sci Rep. 2021 Jan 18;11:1644. doi: 10.1038/s41598-020-80383-1 (PMC7814057; doi:10.1038/s41598-020-80383-1)
Supplement: Supplementary file 1 — Supplementary Information. [file 41598_2020_80383_MOESM1_ESM.docx]

Article

Circulating tumour cells as an indicator of early and systemic recurrence after surgical resection in pancreatic ductal adenocarcinoma

Yejong Park^a,¶^, Hye Ryeong Jun^b,¶^, Hwi Wan Choi^c^, Dae Wook Hwang^a^, Jae Hoon Lee^a^, Ki Byung Song^a^, Woohyung Lee^a^, Jaewoo Kwon^a^, Su Hyeon Ha^a^, Eunsung Jun^a,c^_,_ Song Cheol Kim^a^

a Division of Hepatobiliary and Pancreatic Surgery, Department of Surgery, Asan Medical Center, AMIST, University of Ulsan College of Medicine, Seoul, Republic of Korea

b Biomedical Engineering Research Center, Asan Medical Center, Seoul, Republic of Korea

c Department of Convergence Medicine, Asan Institute for Life Sciences, University of Ulsan College of Medicine and Asan Medical Center, Seoul, Republic of Korea

**^¶^** These two authors contributed equally to this study and should be considered as co-first authors.

Correspondence: Song Cheol Kim; drksc@amc.seoul.kr; Tel: +82-2-3010-3936
 Eunsung Jun; [eunsungjun@amc.seoul.kr](mailto:eunsungjun@amc.seoul.kr) Tel: +82-2-3010-1696

**Figure S1. Overall survival related with recurrence.** (a) Relationship between early recurrence and overall survival. The overall survival was poor when recurrence was observed within 12 months (p < 0.001). The median duration of survival was 14.5 months and 28.0 months, respectively. (b) Relationship between systemic recurrence and overall survival. The overall survival was poor when systemic recurrence was observed (p = 0.010). The median duration of survival was 13.3 months and 28.0 months, respectively.

**
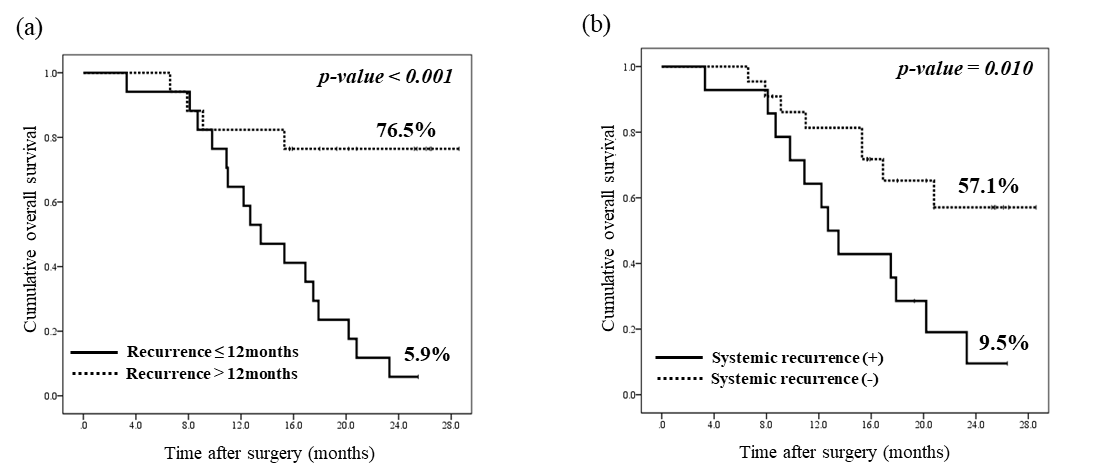
**

**Figure S2. Staining images of detected CTCs.** Detected every single-CTC Images from 17 CTC-detected patients were shown in the order of Merged, CD45, EpCAM/CK, and DAPI. (a) CTC positive cells from 12 patients; CD45(-)/EpCAM-CK(+)/DAPI(+), (b) Double positive cells (grouped as CTC-negative cell) from 5 patients; CD45(+)/EpCAM-CK(+)/DAPI(+). (Front number; patient, back number; cell number)

**(a)**

**
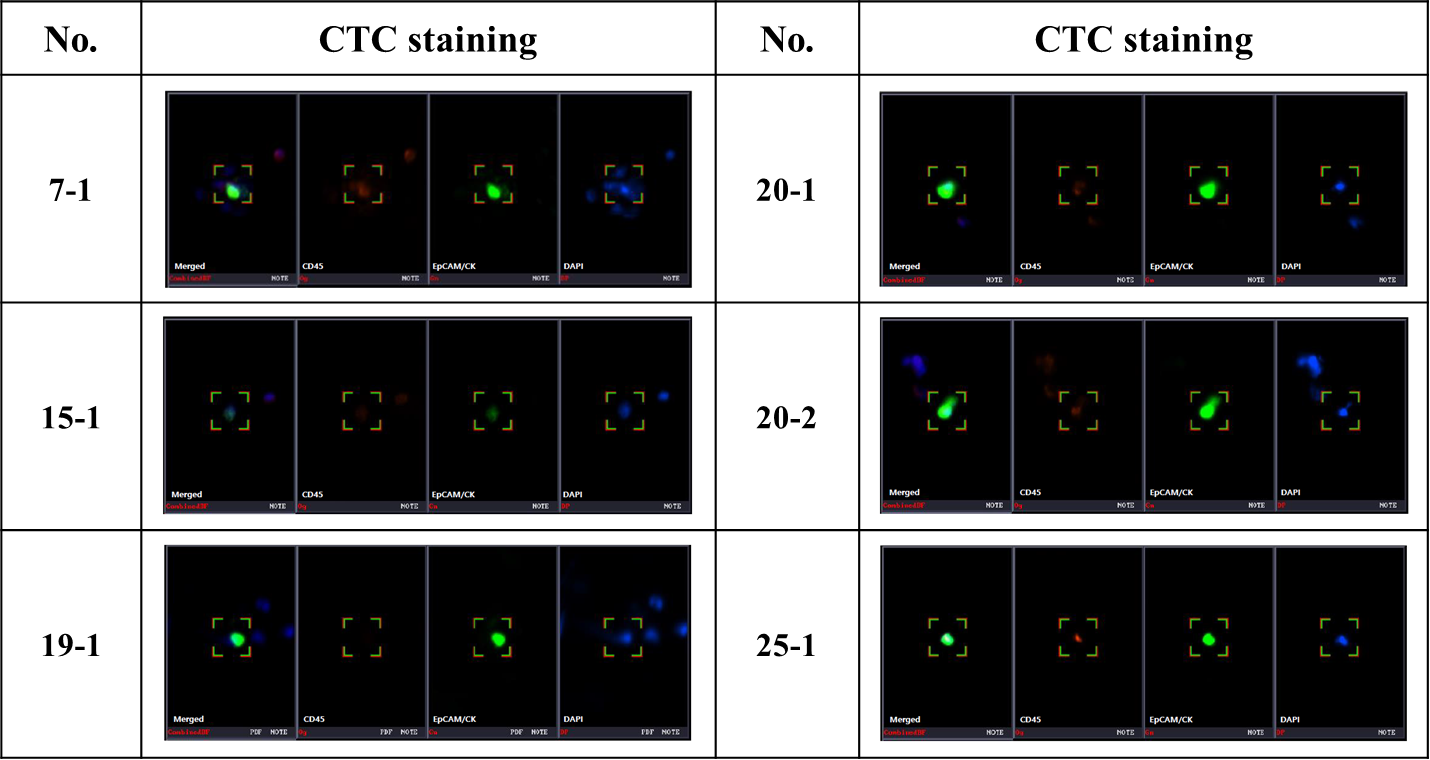

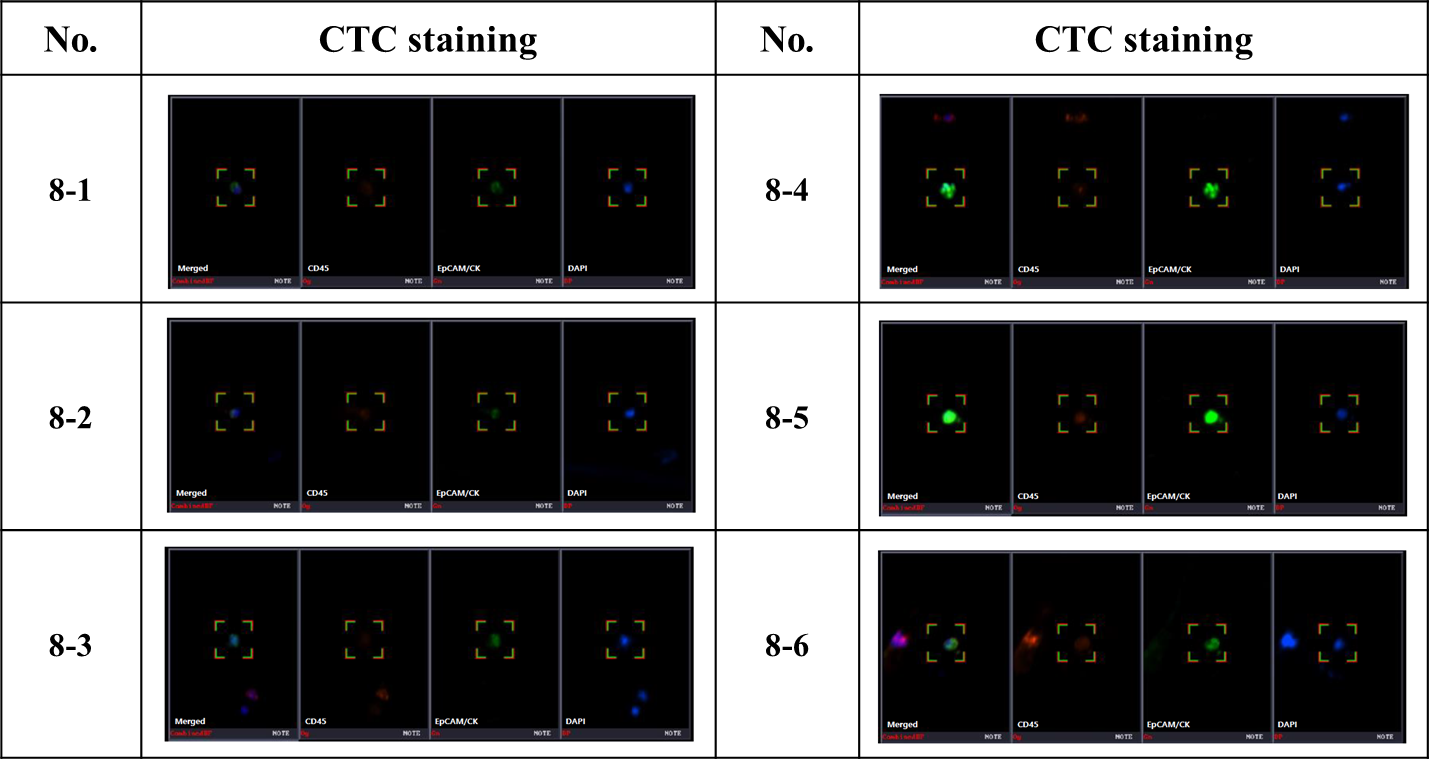
**

**
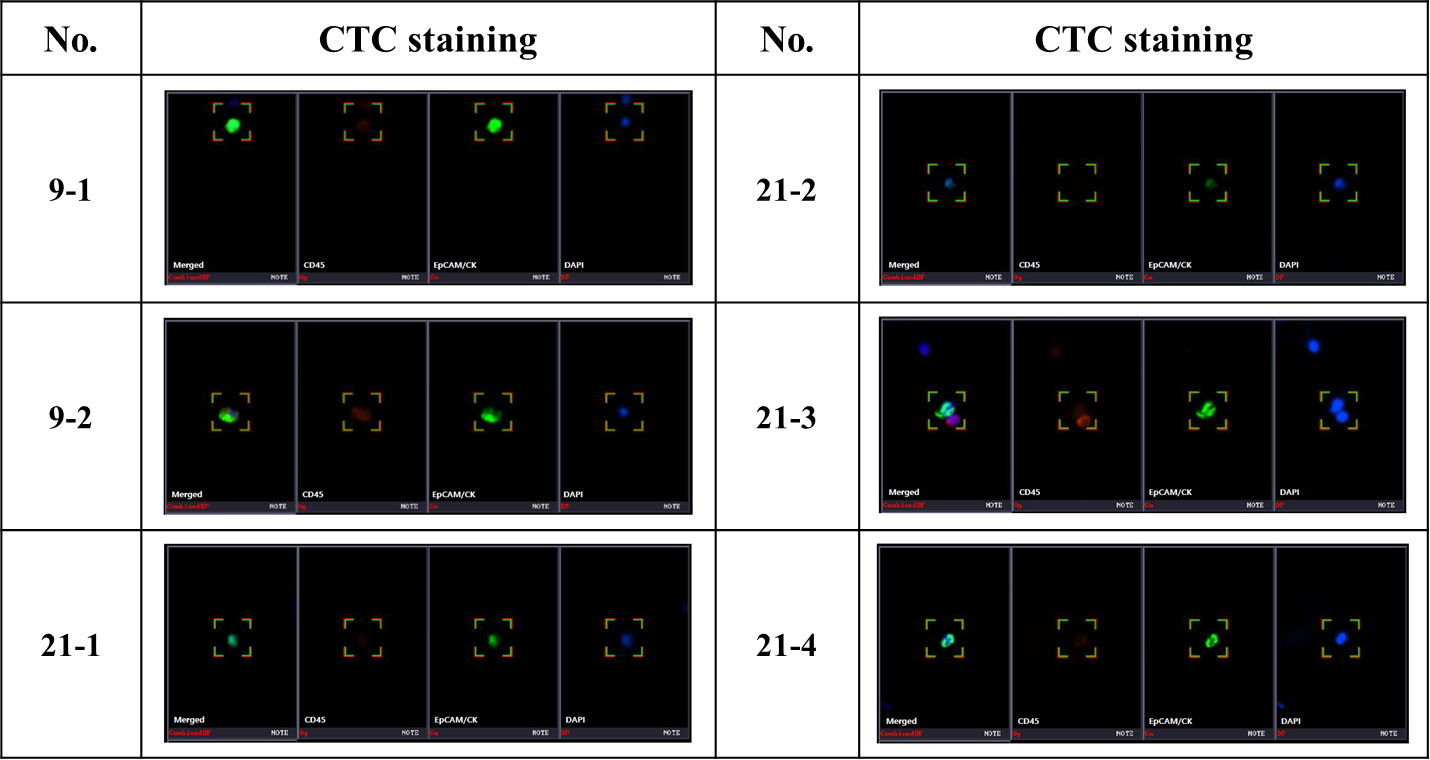

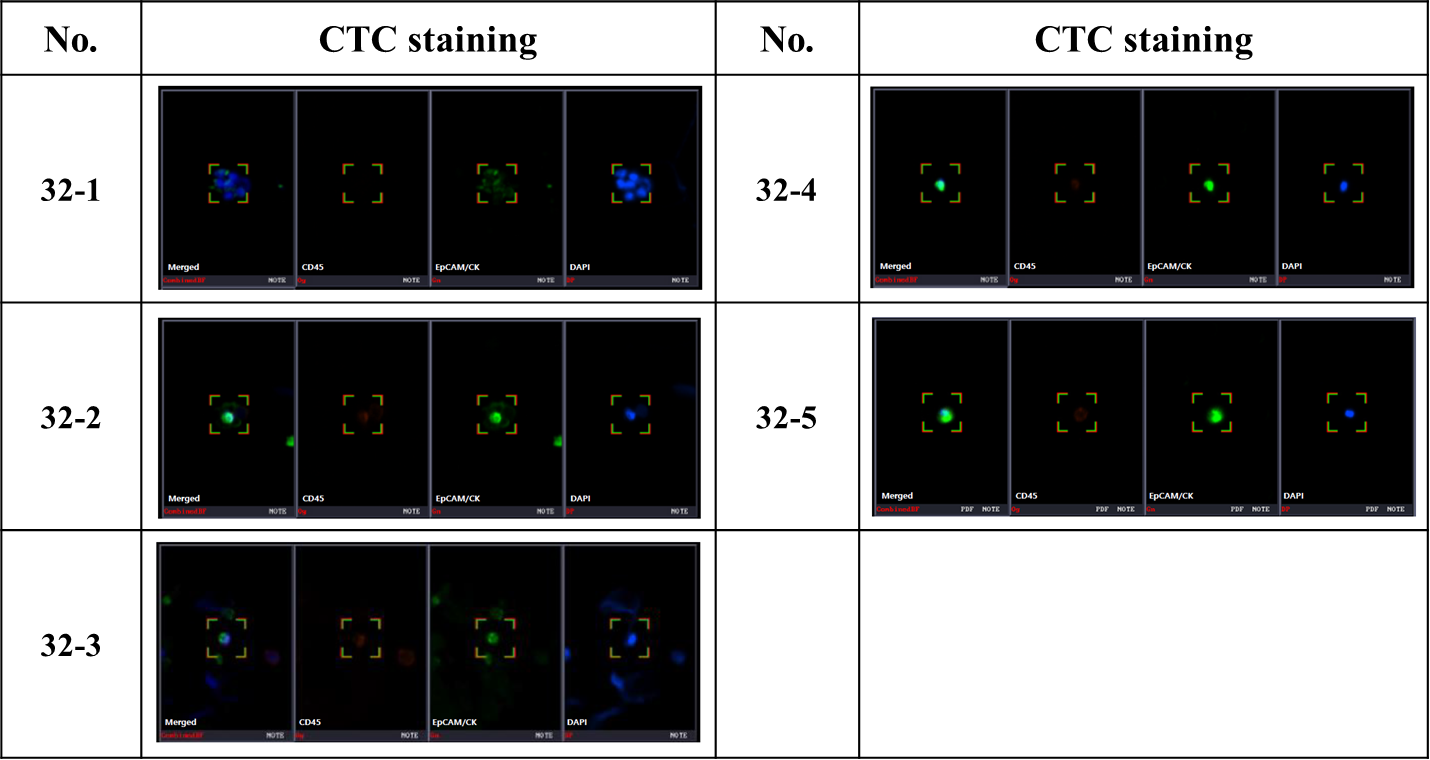
**

**
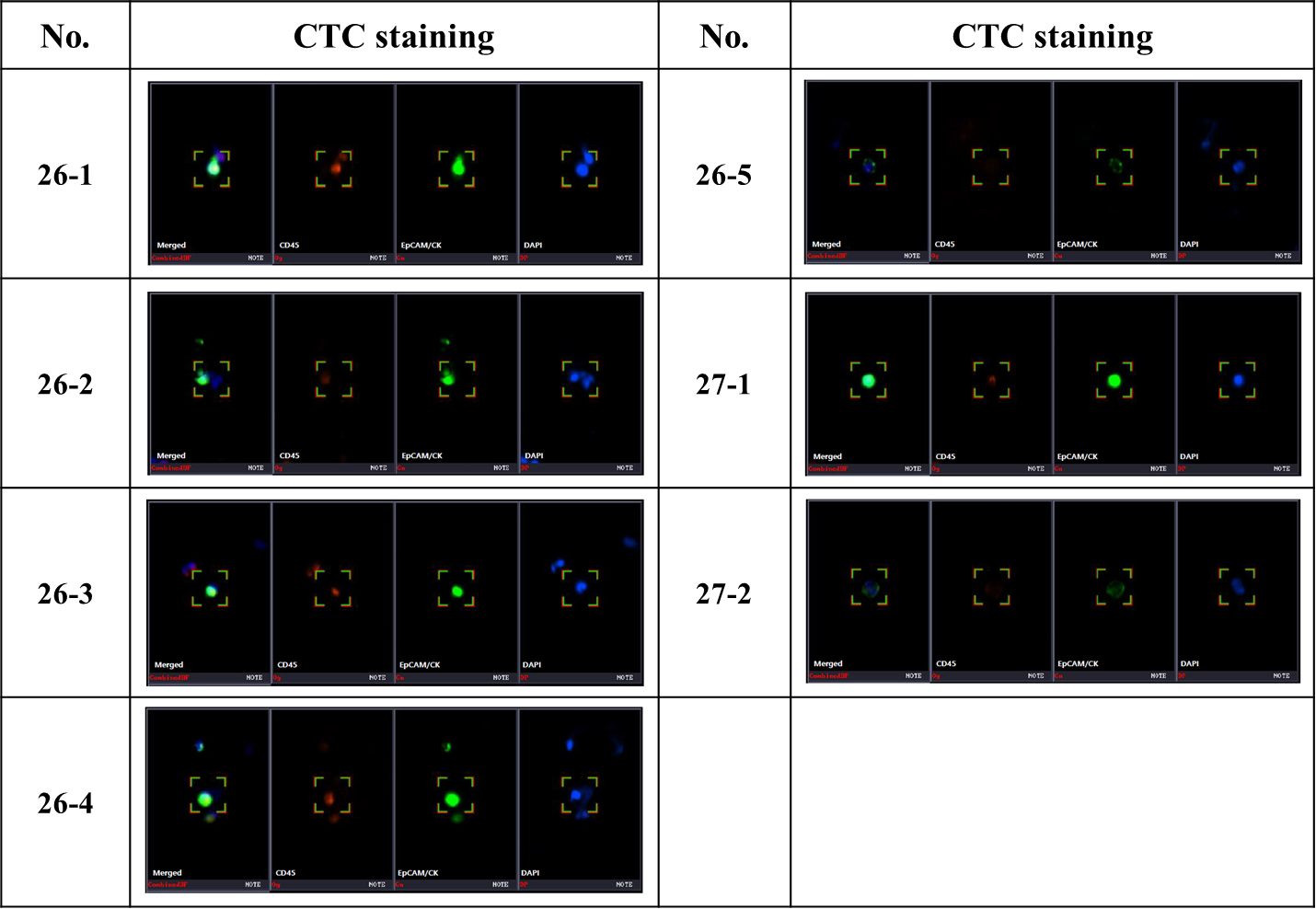

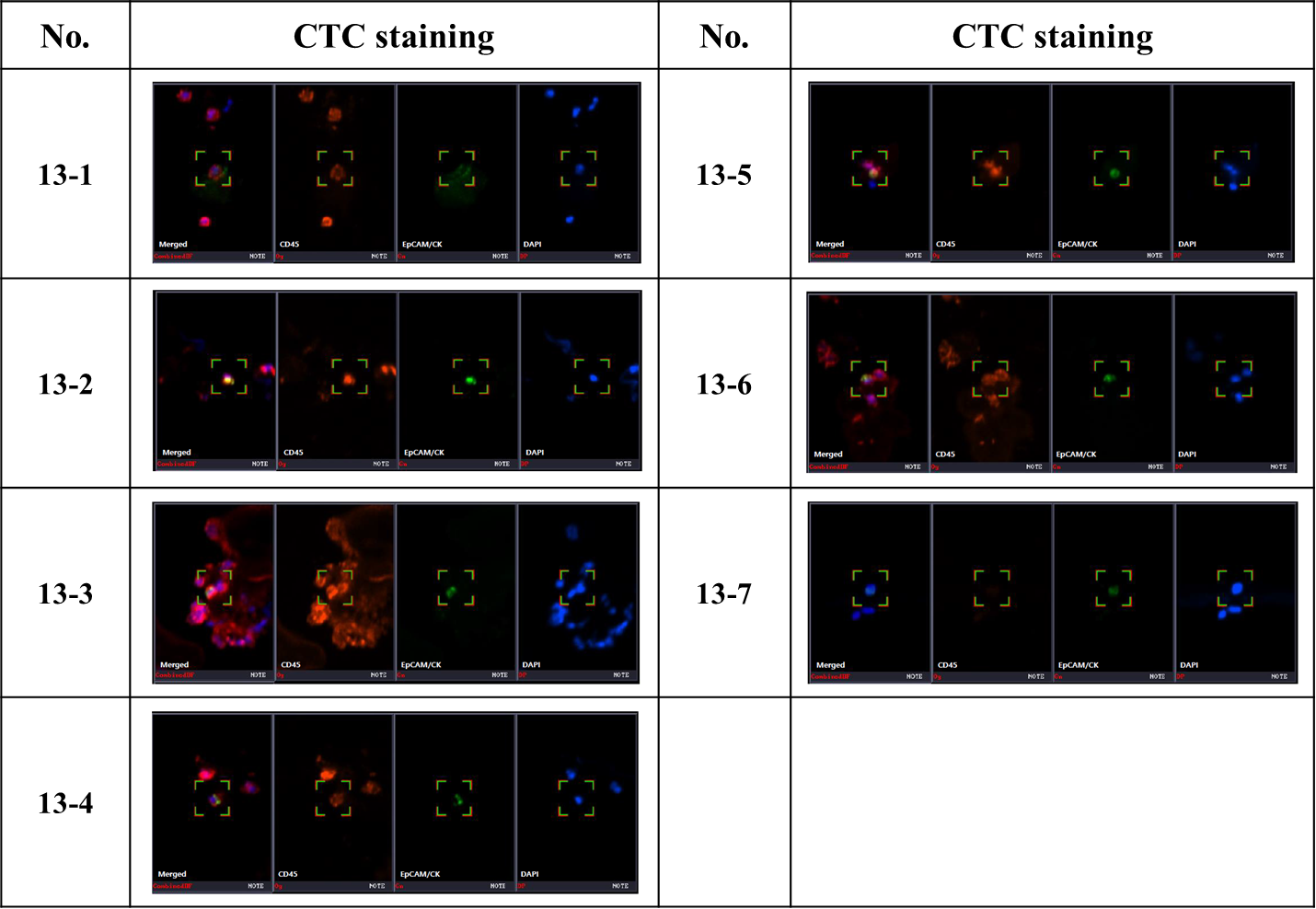
**

**(b)**

**
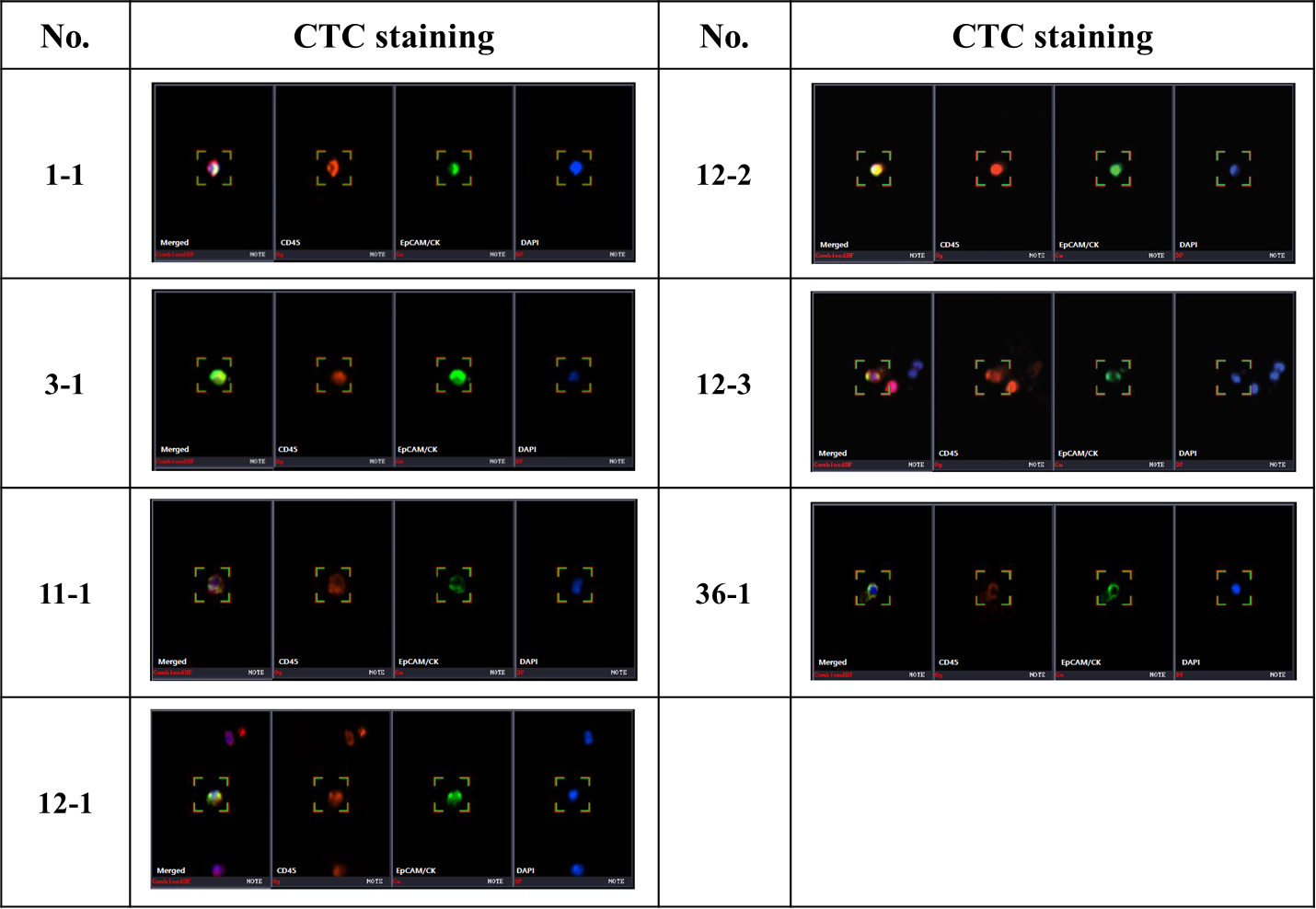
**

**Figure S3. The recurrence pattern and the incidence of cumulative systemic recurrence according to the detection of circulating tumor cells (CTCs).** (a) Systemic recurrence (distant metastasis and peritoneal carcinomatosis) had occurred more frequently than locoregional recurrence (p=0.003). (b) Early recurrence within 12 months was significantly more frequent in the CTC-positive group (p = 0.031).


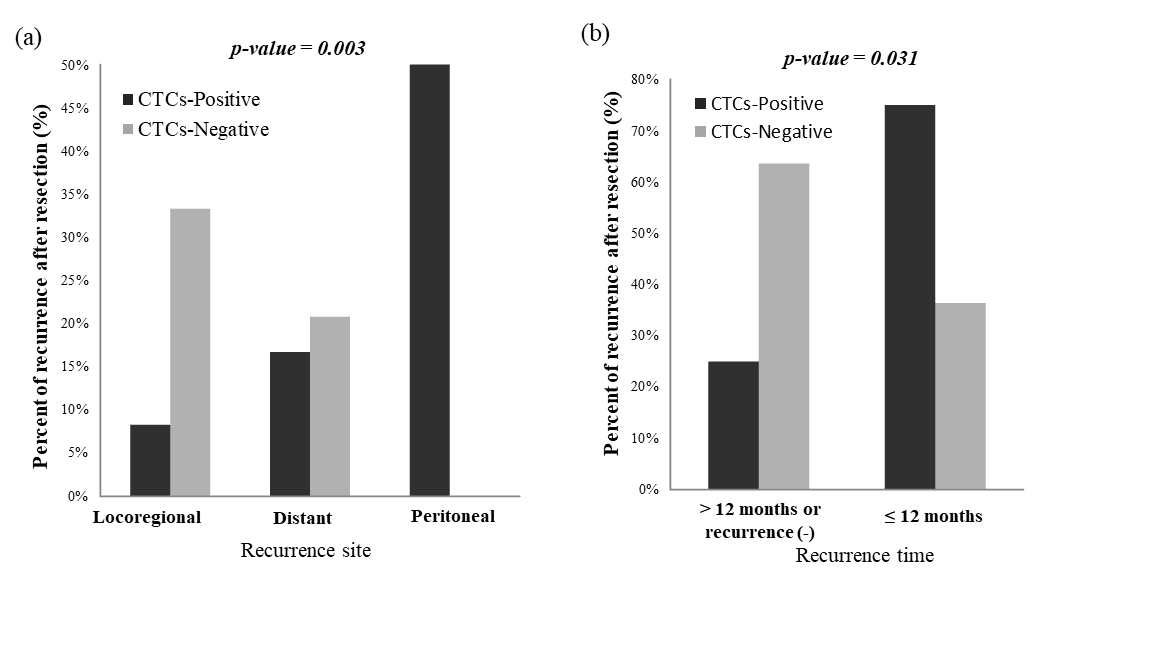


**Table S1. Uni- and Multi-variable analysis of variables affecting overall survival in 36 patients who underwent operation for pancreatic ductal adenocarcinoma.** (ASA classification, American Society of Anesthesiologists physical status classification; CA 19-9, Carbohydrate antigen 19-9; CTCs, Circulating tumour cells; CI, confidence interval)

| Variable | Survival rate | Uni-variable | | | Multi-variable | | |
| --- | --- | --- | --- | --- | --- | --- | --- |
|  |  | **HR**^*^ | **95% CI** | **P-value**^†^ | **HR^1^** | **95% CI** | **P-value**^†^ |
| ASA |  |  |  |  |  |  |  |
| < 3 | 37.8% |  |  | 0.553 |  |  |  |
| ≥ 3 | 50.0% | 0.483 | 0.044~5.350 |  |  |  |  |
| Neoadjuvant Chemotherapy | |  |  |  |  |  |  |
| No | 39.3% |  |  | 0.654 |  |  |  |
| Yes | 29.6% | 1.246 | 0.476~3.260 |  |  |  |  |
| Adjuvant therapy |  |  |  |  |  |  |  |
| No | 60.0% |  |  | 0.539 |  |  |  |
| Yes | 33.9% | 1.586 | 0.364~6.903 |  |  |  |  |
| CA19-9(U/mL) |  |  |  |  |  |  |  |
| < 37 | 36.3% |  |  | 0.570 |  |  |  |
| ≥ 37 | 35.8% | 0.775 | 0.322~1.868 |  |  |  |  |
| CTCs detection |  |  |  |  |  |  |  |
| Negative | 48.6% |  |  | 0.176 |  |  |  |
| Positive | 14.6% | 1.840 | 0.760~4.453 |  |  |  |  |
| R0 resection^‡^ |  |  |  |  |  |  |  |
| Yes | 40.9% |  |  | 0.032 |  |  | 0.194 |
| No | 19.4% | 1.667 | 1.602~4.612 |  | 2.403 | 0.640~9.027 |  |
| Perinerual invasion | |  |  |  |  |  |  |
| No | 50.0% |  |  | 0.805 |  |  |  |
| Yes | 33.5% | 1.168 | 0.340~4.010 |  |  |  |  |
| Lymphovascular invasion | |  |  |  |  |  |  |
| No | 54.7% |  |  | 0.083 |  |  |  |
| Yes | 17.8% | 2.213 | 0.900~5.442 |  |  |  |  |
| TNM stage^¥^ |  |  |  |  |  |  |  |
| < III | 39.1% |  |  | 0.024 |  |  | 0.013 |
| ≥ III | 22.2% | 3.945 | 1.202~12.945 |  | 5.116 | 1.547~14.843 |  |
| Complication |  |  |  |  |  |  |  |
| < grade 3 | 38.4% |  |  | 0.321 |  |  |  |
| ≥ grade 3 | 50.0% | 2.113 | 0.482~9.268 |  |  |  |  |
| Recurrence |  |  |  |  |  |  |  |
| ≥ 12 months | 76.5% |  |  | 0.002 |  |  | 0.007 |
| < 12 months | 5.9% | 5.750 | 1.903~17.378 |  | 4.792 | 1.654~16.454 |  |
| Systemic recurrence | |  |  |  |  |  |  |
| No | 55.7% |  |  | 0.019 |  |  | 0.175 |
| Yes | 9.5% | 2.957 | 1.198~7.302 |  | 2.215 | 0.702~6.986 |  |

^*^ Hazard ratios estimated using Cox regression models excluding possible confounding variables.

^†^ The p-values were calculated using the Cox proportional hazard model.

^‡^ R0 was defined as a distance of <1 mm from the tumour to the resection margin.
^¥^ TNM stage were defined based on AJCC (American Joint Committee on Cancer) 8th edition.
